# Liquid Film Capillary Mechanism for Densification of Ceramic Powders during Flash Sintering

**DOI:** 10.3390/ma9040280

**Published:** 2016-04-11

**Authors:** Rachman Chaim

**Affiliations:** Department of Materials Science and Engineering, Technion-Israel Institute of Technology, Haifa 32000, Israel; rchaim@technion.ac.il; Tel.: +972-4-829-4589

**Keywords:** flash sintering, spark plasma sintering, densification, melting, electric field, electric conductivity, ceramics, invasion percolation

## Abstract

Recently, local melting of the particle surfaces confirmed the formation of spark and plasma during spark plasma sintering, which explains the rapid densification mechanism via liquid. A model for rapid densification of flash sintered ceramics by liquid film capillary was presented, where liquid film forms by local melting at the particle contacts, due to Joule heating followed by thermal runaway. Local densification is by particle rearrangement led by spreading of the liquid, due to local attractive capillary forces. Electrowetting may assist this process. The asymmetric nature of the powder compact represents an invasive percolating system.

## 1. Introduction

Spark plasma sintering (SPS) and Flash sintering (FS) are nowadays considered important processes for rapid densification of ceramic particles to fully dense solids. The two techniques differ by the set-up, the ranges of the applied voltage and the electric current density; however, the physical and chemical processes, and the electric field effects with the ionic/semiconducting ceramics are very similar. In this respect, different densification mechanisms suggested for the enhanced densification kinetics, where the processes at the particle surfaces and interfaces play the dominating role [1,2,3,4,5,6,7,8,9,10,11,12]. Recently, local melting of ceramic particle surfaces further supported the formation of spark and plasma during SPS [13,14,15,16,17]. Therefore, spark and plasma related to discharge of surface charges, accumulated on the non-conducting particle surfaces [18]. Consequently, no spark and plasma were expected in conducting ceramic particles subjected to SPS; the enhanced densification in the later systems is associated with excessive Joule heating at the particle contact points. This latter behavior, where a contiguous particle network provides a percolative path for electric conduction, is tangent to the FS process. Thus, Joule heating followed by thermal runaway are the main current explanations for flash sintering. Yet, densification in FS specimens lacks the corresponding rapid densification mechanism.

Although extensive efforts have been invested towards characterization of the FS parameters and the voltage/current behavior, less attention has been paid to understanding of the involved densification mechanisms, with respect to the observed microstructure. In this regard, three major problems exist: first, most of the analyses relate the actual local process temperature to the temperature of the specimen, either technically measured (thermocouple, pyrometer), or calculated from the average value of the current (*i.e.*, black body calculation from electrical energy dissipation), or indirect measurements (*i.e.*, thermal expansion calibrations [19]). Consequently, the actual process temperature at the particle contacts is uncertain, and its estimation may vary by hundreds of degrees from work to work [8,19,20,21]. Second is the assumption that sintering takes place at once throughout the specimen. This assumption originates from the lack of interrupted FS experiments, or incorrect interpretation of the resulting microstructures. The third is the assumption about solid state sintering during the FS process. Simple calculation of the diffusion distances necessary for mass transport, using the ionic diffusion coefficients fail to explain the densification within the typical time intervals of flash sintering by solid state sintering.

The yield stress and electrical conductivity, and their temperature dependence, are the main properties that determine the conditions of the field effects, *i.e.*, at what conditions spark and plasma will form in a given non-conducting granular compact. These properties are used to construct plastic-deformation plasma-formation temperature window diagrams [13,14,18,22], from which one can deduce the SPS pressure-temperature schedule appropriate for enhanced densification. This introduces a new region of enhanced densification by plasma, with a transient nature, into the conventional densification mechanism maps. The relative location of this new region is determined by the plasma-formation temperature window; its position is expected to shift due to the particle size dependence of the yield strength and the surface conductivity, as well as the value of the applied pressure. Therefore, this transient state of rapid densification in SPS was previously termed “surface softening”, due to the uncertainty concerning the nature of the liquid at the particle surfaces [23].

Following several reported microstructures of ceramics densified via FS, one can observe crystal growth from remnant of liquid phase [24], crystal growth from vapor [25], and curved boundaries, which are characteristic of a liquid presence during sintering [26]. Furthermore, the local nature of melting/reaction was confirmed by the islands in the partially reacted microstructure [9]. Several works also reported the formation of local liquid as well as heterogeneous microstructure during flash sintering [9,25]. Therefore, in this paper I will evaluate the formation of liquid at the particle contacts during FS and its consequences upon densification kinetics and microstructure via capillary forces.

## 2. Analysis and Discussion

### 2.1. Spark Plasma Sintering

The formation of spark and plasma depends on many material and process parameters, and takes place as a transient phenomenon during the densification. The charged particle surfaces at the compact cavities act as sources for spark and plasma [18], when they attain certain conditions for percolation of the electric current. The ignited plasma enhances densification under minimal applied pressure, via particles sliding aided by a “softened layer” (here will be treated as a liquid film) at their surfaces. The sudden increase in density and particle connectivity suppresses further expansion of the plasma; hence, it represents a local process with transient non-equilibrium character. Therefore, plasma region in the deformation mechanism map is a sort of “ladder” for climbing to higher densities within extremely short durations.

When spark and plasma increase the surface temperature so that a liquid film forms at the particle surfaces, the particles can sinter together in the absence of external pressure, or slide over each other by viscous flow, in the presence of an external pressure [13]. The resistance to mutual sliding of the two solid particles covered with a liquid film depends on the relative thickness of the liquid film compared to the particles radius [27]. Jagota and Dawson [27] treated this problem and showed that under certain conditions (*i.e.*, liquid film thickness to particle radius ratio higher than 0.2) the system behaves if no solid particles exist, *i.e.*, densification may take place by simple viscous sintering. However, such liquid layer thicknesses are unseen, neither in SPS, nor in FS, and I restrict the discussion to melting of a few atomic layers (*i.e.*, film) at the contact points between particles. 

### 2.2. Flash Sintering

The specimen set-up in FS technique assumes compacted green powder through which the electric current passes via external electrodes, while the specimen is heated within the furnace. The green strength assures particle contiguity and available path for percolation of the electric current at the flash temperature. The main event of flash is a non-linear increase of the current at the onset flash temperature. Du *et al.* [10] attempt to explain this non-linearity as an artifact due to the estimation method of the specimen temperature. However, their measurements also refer to the average temperature instead of a local temperature in the specimen. Such non-linearity in the local current at the particle contacts may exist and explain the rapid sintering and densification, as shown below.

#### 2.2.1. Local Capillary Forces

Recent FS works support the thermal runaway model as a dominating process at the flash set point [8,10,28]. Several significant processes may take place if the actual local temperature at the contact point surpasses the melting point of the solid particle at contact due to the Joule heating. First, the similar composition of the melt and the solid particle from which it melted leads to very low solid-liquid interfacial energy, hence full wetting of the solid particles by the melt at the contact point. The capillary forces associated with such a liquid layer depend on the solid particle size. For 100 nm Al_2_O_3_ nano-particles (in diameter) with liquid-vapor surface tension of 665 mJ∙m^−2^ in air [29], attractive capillary force of ~27 MPa is estimated. This capillary force is of the same order of the pressures applied during the SPS and hot pressing, and is high enough to attract the adjacent particles and lead to their local rearrangement and compaction.

Another aspect of wetting which is worth noting is the occasions when local melting and wetting cause the formation of a gap (previously a contact) between the particles. In such a case, the presence of high local electric fields over the micrometric or nanometric gaps can lead to electrowetting [30] of the ionic melt, and affect both the liquid dihedral angle as well as the spreading degree of the liquid on the particle surfaces.

#### 2.2.2. Local Electric Conductivity

Let us assume that the green compact of ceramic powder is heated and subjected to increasing current density at constant voltage. Such a system is composed of different resistances (particles and contact points) and capacitances (particle gaps). Once an appropriate electrical conductivity is gained, the higher electric resistivity at the particle contacts preferably consumes the current for the Joule heating. The local heating at the contact point increases the local temperature, and consequently the local electric conductivity (ionic and electronic), due to the negative temperature coefficient of resistivity. This process has an autocatalytic effect, which may eventually lead to local melting at the contact points. Formation of melt at the contact point also has significant implications on the FS process, which have been underestimated until now, if not neglected. The ionic conductivity in many oxides (*i.e.*, BeO, Al_2_O_3_, Sc_2_O_3_, ZrO_2_, Y_2_O_3_) experiences an abrupt jump at melting, where the conductivity in the melt may increase by two to four orders of magnitude compared to the crystalline state [31,32]. The conductivity in pure oxide melts is controlled by cations, when ionic bonds dominate (*i.e.*, MgO, CaO, Li_2_O, Al_2_O_3_), and by electrons, when significant covalent bonding exists, and leads to semiconducting behavior (*i.e.*, Bi_2_O_3_, CuO, MnO, TiO_2_, V_2_O_3_). The electric conductivity (*σ*) in such melts depends on the bond strength, as well as on the size of the structural units moving within the melt, hence the melt viscosity (*η*). The product of the electric conductivity by the viscosity for a given ion is constant and expressed by Walden’s rule [33]:

(1) $$\sigma \cdot \eta =n_{i,melt}\frac{{\left(Z\cdot e\right)}^{2}}{3\pi d}=constant$$

where *n_i,melt_* is the number of the *i*-th ion per unit volume when the transport number is unity, *Z* is the ion charge, *d* is the ion diameter, and *e* is the unit charge of the electron. This model assumes that the conducting species are spherical ions that are in steady state flow. Nevertheless, viscosity models assume flow of structural units and associate species within the ceramic melts, rather than single ions. Using the viscosity of pure ceramic melts and their volume, one can estimate fairly well the electric conductivity at the corresponding temperature.

Here I will limit the discussion to the case of pure Al_2_O_3_, for which there is extensive reliable physical and chemical data in the literature and has been densified by flash sintering [34]. The structural units in alumina melt are AlO_1.5_, which represent a typical oxygen octahedron around the Al^+3^ cation [35]. Assuming partial ionic conductivity, *i.e.*, transference number 0.5, via diffusion of aluminum octahedron in the melt, the equivalent ionic diameter using the octahedron volume (as a sphere) is 2.397 nm. These assumptions lead to the more conservative calculation of the electrical conductivity (*i.e.*, lower values). In addition, the number of the ions per unit volume in the melt decreases compared to that of the crystalline solid, due to the decrease in the melt density. Therefore, the number of ions per unit volume in Equation (1) normalized by:

(2) $$n_{melt}=n_{solid}\frac{\rho_{melt}}{\rho_{soild}}$$

where *ρ_i_* and *n_i_* are the density, and the number of the aluminum ions per unit volume, respectively, in either the melt or the crystalline solid.

**Figure 1:**
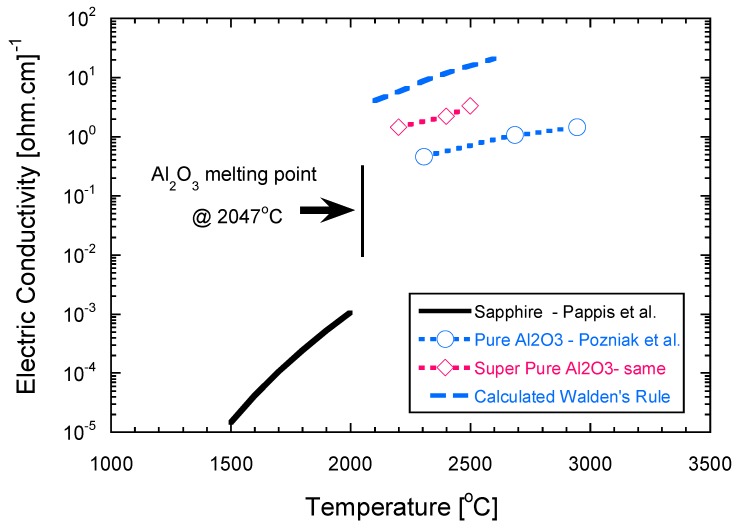
Electric conductivity of Sapphire crystal (black solid line) and alumina melt (blue dashed line) calculated from Walden’s rule. The measured experimental values for pure (blue open circles) and super-pure alumina (red open diamonds) melts presented for comparison. Melting leads to increase in the electric conductivity by two to four orders of magnitude.

Combining Equations (1) and (2), and using the following data for alumina: *n_solid_* = 6, *ρ_solid_* = 3.98 g·cm^−3^, *d* = 2.397 nm, *e* = 1.602 × 10^−19^ coul, the electric conductivity of alumina was calculated from its melt viscosity [35] and its melt volume change [36] *versus* temperature. These data, with some experimental conductivity values measured in air [37,38], are presented in Figure 1. The calculated electric conductivity values (blue dashed line) are higher only by one order of magnitude from the experimentally measured values of super pure Al_2_O_3_. Overall, melting of alumina was followed by increase of electric conductivity at the melt by at least two orders of magnitude. Therefore, local melting at the contact points may increase the local current density by two orders of magnitude. The probability for local melting depends on the relative local conductance of the contact points available within the green compact. The smaller the contact diameter, the higher the current density through it, hence the higher the probability for melting. This should also lead to lower onset temperature for the flash, consistent with the reported data on the particle size effects [39]. Consequently, local melting should first take place at the loci of smaller contacts (*i.e.*, smaller particles), within the green compact.

Since the liquid between the two contacting particles assumed to wet both particles, further melting should take place at the contacts of larger particles. Thus, the local melting progress is hierarchical, which assures the preservation of the local melt, as long as other percolative paths for the current flow exist. As was mentioned above, formation of a local melt leads to capillary forces high enough for local rearrangement of the surrounding particles. The local electrical resistance after melting, wetting, and rapid local densification is free of contact resistance, and falls to low values of the melt resistivity. Therefore, further dissipation of the electrical energy by the Joule heating will take place at other solid contacts having higher electrical resistances. This change in the local conductivity provides the conditions needed for promotion of this liquid assisted rearrangement and densification mechanism by capillary forces at different loci throughout the compact. The asymmetric nature of the powder compact subjected to electric current and connected to two different electrodes (*i.e.*, Cathode and Anode) represents an invasive percolative system [40]. In addition, the ceramic particles are most often characterized by a fractal character, which may change their electrical response. I will treat these aspects in a future paper since the present densification model of liquid film capillary is still valid.

#### 2.2.3. Local Volume Change

**Figure 2:**
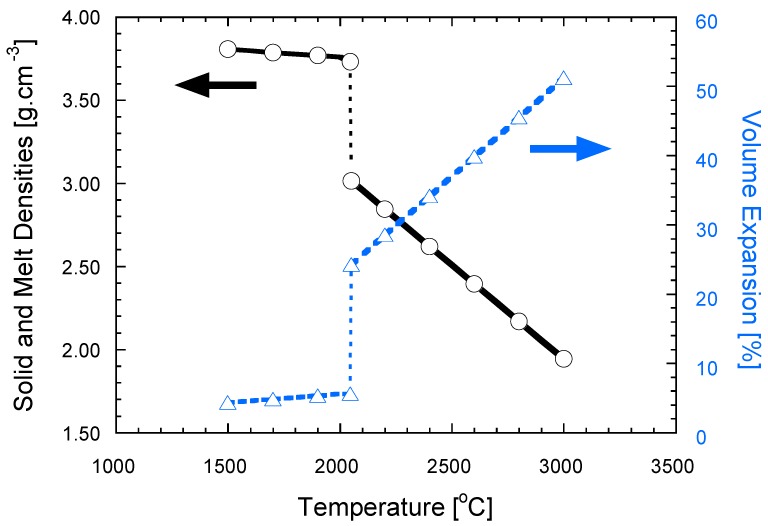
Densities of crystalline alumina and its melt (solid black line) *versus* temperature exhibits discontinuous decrease at the melting temperature. The corresponding increase in the specific volume expansion with temperature presented by the dashed blue line. Data for the melt density used from reference [36].

Local melting also leads to significant increase in the specific volume, expressed by a decrease in the melt density, compared to that of the crystalline solid. The lattice parameters of Sapphire are c = 1.29915 nm, a = 0.47592 nm, using the hexagonal notation of the rhombohedral lattice. The thermal expansion coefficients of Sapphire above 1500 °C are almost constant, with values of *α_c_* = 9 × 10^−6^ °C^−1^ and *α_a_* = 8 × 10^−6^ °C^−1^ [41]. Using the lattice parameters of Sapphire and its thermal expansion coefficients, the changes in density (black solid line) and the specific volume (dashed blue line) were calculated and shown in Figure 2. The corresponding data for the melt density and its specific volume are also plotted in Figure 2, using data from the literature [36]. The specific volume in Al_2_O_3_ increases by ~20% at the melting temperature; further linear increase observed with temperature, albeit with much higher gradient than in the solid (dashed blue lines in Figure 2). Once melting takes place at the contact point, the local volume increase provides a liquid meniscus necessary for wetting of the adjacent solid particles, and aids their local rearrangement and densification.

The discontinuous increase in both electrical conductivity and specific volume with melting are inherent physical properties of ceramic crystals. Therefore, all ceramic powder compacts subjected to an electric field are prone to flash sintering, once critical flash conditions attained. The flash onset condition is mainly controlled by the amount of the applied electric power density [28], and leads to local melting at the particle contacts. The non-linear electric conductivity is associated with this local melting. In this respect, simulations of flash sintering confirmed the thermal runaway to be a consequence of the temperature dependent resistivity [42]. Narayan proposed a model for grain boundary melting, albeit for description of grain growth during flash sintering [43]. Finally, striking similarities exist between the flash sintering kinetics of sub-micrometer pure alumina [44] and that of the same powder with 2 mol% liquid forming additives, subjected to conventional sintering [45]; this indicates the important role of the liquid phase during flash sintering.

## 3. Conclusions

A model of liquid-film capillary was introduced as a mechanism for the rapid densification during flash sintering. The thermal runaway due to the preferred Joule heating at the particle contacts leads to local melting at these loci, followed by particle wetting. The attractive capillary forces associated with this liquid film lead to particle rearrangement hence to densification. The rapid densification aided by the local increase both in the specific volume and in the electric conductivity due to the melt at the contacts. The contacts melt in a random hierarchical manner and the process has an asymmetric nature. The overall process is a critical phenomenon and modeled by an invasion percolation. 

## Abbreviations

The following abbreviations used in this manuscript:

SPS	Spark Plasma Sintering
FS	Flash Sintering
